# Activity-dependent synaptic competition and dendrite pruning in developing mitral cells

**DOI:** 10.3389/fncir.2025.1541926

**Published:** 2025-01-27

**Authors:** Takeshi Imai

**Affiliations:** Department of Developmental Neurophysiology, Graduate School of Medical Sciences, Kyushu University, Fukuoka, Japan

**Keywords:** synapse elimination, synaptic competition, dendrite pruning, mitral cell, olfactory bulb, spontaneous activity, NMDA receptor, RhoA

## Abstract

During the early postnatal period, neurons in sensory circuits dynamically remodel their connectivity to acquire discrete receptive fields. Neuronal activity is thought to play a central role in circuit remodeling during this period: Neuronal activity stabilizes some synaptic connections while eliminating others. Synaptic competition plays a central role in the binary choice between stabilization and elimination. While activity-dependent “punishment signals” propagating from winner to loser synapses have been hypothesized to drive synapse elimination, their exact nature has remained elusive. In this review, I summarize recent studies in mouse mitral cells that explain how only one dendrite is stabilized while others are eliminated, based on early postnatal spontaneous activity in the olfactory bulb. I discuss how the hypothetical punishment signals act on loser but not winner dendrites to establish only one primary dendrite per mitral cell, the anatomical basis for the odorant receptor-specific parallel information processing in the olfactory bulb.

## Introduction: activity-dependent remodeling of neuronal circuit

Neurons in the brain communicate with each other through synapses. Synaptic connections are formed from late embryonic to early postnatal periods. Classically, the mechanisms of the circuit assembly have been understood on the basis of two distinct phases in vertebrates. Initially, molecular guidance cues and synaptic organizers defined by the genetic programs navigate axons, dendrites, and synapse formation (Kolodkin and Tessier-Lavigne, 2011; Siddiqui and Craig, 2011; Sanes and Zipursky, 2020). At this stage, the connectivity is established based on the limited set of molecular guidance cues, leaving a lot of miswiring at finer scales. Later, activity-dependent remodeling fine-tunes the exuberant connections to form more precise and functional neuronal circuits. In this process, some connections are strengthened, while others are eliminated. Neurons that initially receive heterogeneous synaptic inputs gradually become tuned to specific sets of inputs. In this way, neurons in sensory circuits establish a discrete receptive field. In recent years, the interplay between genetically programmed and activity-dependent mechanisms has also been reported. For example, spontaneous, rather than sensory-evoked, neuronal activity plays a critical role in the earlier activity-dependent process (see below). It is also recognized that activity sometimes influences gene regulation for guidance molecules (Imai and Sakano, 2008; Cheng et al., 2022). Nevertheless, circuit development proceeds in a stepwise fashion, with the initial formation of approximate connectivity followed by an activity-dependent fine-tuning process (Goodman and Shatz, 1993).

The role of activity-dependent refinement was revealed by the seminal works by Hubel and Wiesel in the visual system (Wiesel and Hubel, 1963b, 1963a, 1965b, 1965a). In the visual cortex, monocular deprivation leads to a weakening of inputs from the closed eye and a strengthening of inputs from the open eye, known as ocular dominance plasticity. In the lateral geniculate nucleus of the thalamus, many neurons initially receive inputs from both the right and left eyes. However, through an activity-dependent refinement process, they eventually establish inputs from only one eye (Penn et al., 1998). The synapse elimination is not simply long-term depression (LTD) based on weak synaptic inputs; the relative magnitude of the inputs (e.g., right versus left eye) is important for synapse stabilization versus elimination (Goodman and Shatz, 1993).

The timing of the synaptic inputs is also critical for circuit refinement. It is known that the blockade of NMDA receptors (NMDARs) impairs the refinement process (Cline et al., 1987; Kleinschmidt et al., 1987). Carla Shatz proposed that *“cells that fire together are wired together”* based on Hebbian rule of plasticity (Hebb, 1949; Shatz, 1992). This means that neurons receiving strong and correlated synaptic inputs stabilize these synapses based on LTP-like mechanisms. She also proposed *“out-of-sync, lose your link,”* explaining that neurons receiving weaker and decorrelated inputs lose these connections. Indeed, subsequent studies have demonstrated that synchronized synaptic inputs lead to the stabilization of synapses, whereas desynchronized inputs lead to the elimination of weaker inputs (Zhang et al., 2012; Matsumoto et al., 2024). It is important to note that the synapse elimination is the result of an active elimination process, not just a failure to stabilize the synapses. In the remodeling process, synapse formation/stabilization and elimination are equally important. However, compared to the mechanisms of circuit formation/stabilization, much less is known about synapse elimination. In particular, the instructive signals for synapse elimination are mostly unknown.

## Synapse elimination in model systems and hypothetical “punishment signals”

**Figure 1:**
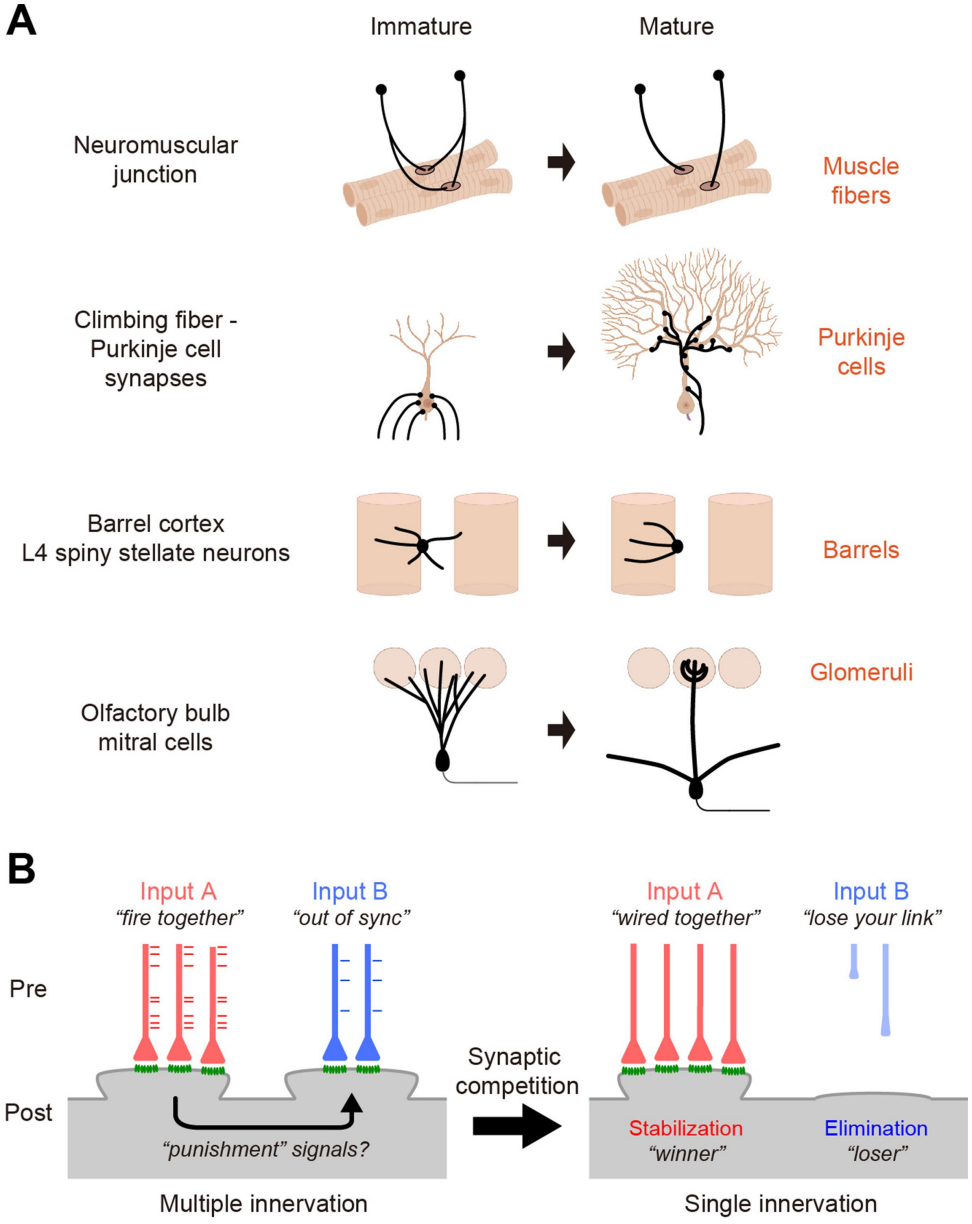
Synaptic competition in model systems. **(A)** At neuromuscular junction and climbing fiber—Purkinje cell synapses, axons from different neurons compete for one postsynaptic cell (muscle fibers and Purkinje cells, respectively). In L4 neurons in the barrel cortex and mitral cells in the olfactory bulb, dendrites connecting to a single presynaptic input are stabilized, while those receiving other types of inputs are eliminated. **(B)** Synapse elimination in these models is the result of synaptic competition in postsynaptic cells. Different types of synaptic inputs compete with each other. The red and blue tick marks indicate the spike timing at the red and blue axons, respectively. Eventually synapses that receive strong and synchronized inputs are stabilized and all the others are eliminated. It has been hypothesized that “punishment” signals from the prospective winner synapses to loser synapses lead to synapse elimination (Lichtman and Colman, 2000). We obtained a permission from BioRender for the use of **(A)**.

While the visual system is one of the most extensively studied model systems, developmental synapse elimination has been studied in several model systems in mammals (Figure 1A). In some systems, axons compete for one postsynaptic target. In other systems, dendrites from the same neuron compete with each other to establish one type of synaptic input per neuron. Thus, synaptic competition is the driving force behind the elimination process.

The neuromuscular junction is also a classic model of synaptic competition (Sanes and Lichtman, 1999). In newborn mice, a neuromuscular junction is innervated by multiple axons from motor neurons. However, all but one axon are eliminated in an activity-dependent manner, as nicely shown by *in vivo* imaging (Walsh and Lichtman, 2003). This is likely to be the basis for fine control of motor movement. Relative synaptic inputs are important for synapse elimination (Dan and Poo, 1992; Buffelli et al., 2003), suggesting a role for synaptic competition (Lichtman and Colman, 2000).

Similarly, developmental axonal elimination is known in the climbing fiber—Purkinje cell synapses (Watanabe and Kano, 2011). In the newborn animals, multiple climbing fibers form synapses on one Purkinje cell. However, during postnatal development, only one axon forms strong synapses on dendrites, while others are gradually eliminated, to establish only one climbing fiber per Purkinje cell (Hashimoto et al., 2009; Carrillo et al., 2013). This is the anatomical basis for the precise feedback of the error signals that is critical for LTD-based tuning of parallel fiber inputs to a Purkinje cell.

The mouse barrel cortex has become an excellent model system for dendrite remodeling using mouse genetic tools. Layer 4 in the barrel cortex consists of the barrel hollow and septa regions, of which the hollow receives whisker touch information. Layer 4 spiny stellate neurons located at the periphery of the hollow (L4 neurons, hereafter) initially extend their dendrites in all directions, encompassing multiple hollows and septa; however, during the early postnatal period, the dendrites of a single L4 neuron become all confined to a single barrel hollow (Erzurumlu and Gaspar, 2012; Mizuno et al., 2014). This is the result of dendritic remodeling based on the patchwork pattern of spontaneous activity derived from the trigeminal ganglion (Mizuno et al., 2018; Banerjee et al., 2022). In this case, a few dendrites connected to the same whisker inputs are stabilized, while those connected to other whisker and septa regions are eliminated. As a result, the receptive field of L4 neurons will be tuned to only one whisker. As in the visual system, NMDARs play a critical role in the remodeling process (Iwasato et al., 2000; Espinosa et al., 2009; Mizuno et al., 2014).

Although axons are eliminated in some examples (e.g., neuromuscular junctions and climbing fiber – Purkinje cell synapses), this does not mean that axons directly compete with each other. It is considered that different synaptic inputs are weighted based on the spatiotemporal input to the postsynaptic cells. Thus, the synaptic competition occurs in the postsynaptic cell (Figure 1B). The elimination of axons may be secondary to the elimination of postsynapses. How then is the *“fire together, wire together”* and *“out-of-sync, lose your link”* achieved at a postsynaptic cell? It has been hypothesized that strong synaptic inputs to the prospective “winner” synapses triggers “punishment” signals that propagate to the “loser” synapses, leading to synapse elimination (Lichtman and Colman, 2000). However, the exact nature of the punishment signals remains to be determined (Nagappan-Chettiar et al., 2023).

## Dendrite pruning in mitral cells and “one mitral cell – one glomerulus” rule

To understand the mechanisms of developmental synaptic competition, mitral cells in the olfactory bulb became another useful model system. The characteristic morphology of mitral cells was first described by Camillo Golgi and Santiago Ramon y Cajal in the late 19th century (Shepherd et al., 2011). A typical mitral cell has a single primary dendrite connected to just one glomerulus and several lateral dendrites that extend within the external plexiform layer of the olfactory bulb. However, the functional role of this characteristic morphology was not fully recognized until the discovery of odorant receptors (ORs) by Buck and Axel (1991) and their characterization in the olfactory system (Mori et al., 1999).

**Figure 2:**
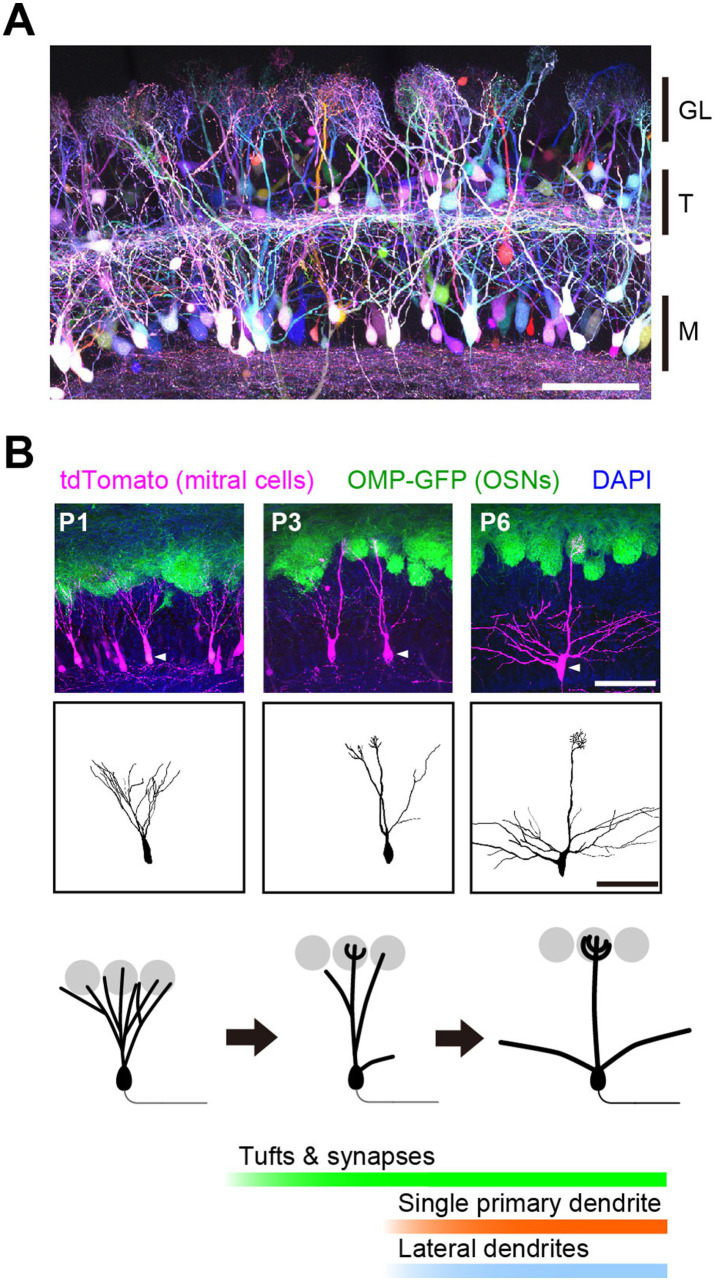
Developmental dendrite pruning of mitral cells. **(A)** Mitral and tufted cells in the olfactory bulb at P7. OSN inputs to one glomerulus are conveyed to 20–50 mitral/tufted cells. Each mitral/tufted cell connects a single primary dendrite to a glomerulus and receives synaptic input from only one type of OSNs. Scale bar, 100 μm. GL, glomerular layer; T, tufted cells; M, mitral cells. Image from Sakaguchi et al. (2018). **(B)** Early on, a mitral cell typically extends multiple dendrites to multiple glomeruli. During development, they prune all but a single primary dendrite that connects to a single glomerulus. Tufted dendrites and postsynaptic structures begin to form just before dendrite pruning. Scale bar, 100 μm. Modified from Fujimoto et al. (2023).

There are ~1,000 functional ORs in the mouse. Each olfactory sensory neuron (OSN) expresses only one type of OR in a mono-allelic manner, known as “one neuron—one receptor” rule (Serizawa et al., 2004). OSNs expressing the same type of OR converge their axons to the same set of glomeruli, known as “one glomerulus—one receptor” rule. As a result, odor information detected by ~1,000 types of ORs is represented by the spatiotemporal activity of ~1,000 sets of glomeruli in the olfactory bulb (Mori and Sakano, 2011). In each glomerulus, OSN inputs are conveyed to 20–50 mitral/tufted cells, of which 5–20 are mitral cells (Sosulski et al., 2011; Ke et al., 2013; Figure 2A). The morphology of mitral cells indicates that each of mitral cell receives excitatory synaptic inputs from only one type of OSNs representing a single type of ORs, which can be referred to as the “one mitral cell – one receptor” rule. These three rules ensure the parallel processing of odor information with ~1,000 channels (Imai, 2014).

The discrete connectivity of mitral cell dendrites is a result of developmental remodeling (Malun and Brunjes, 1996). Early in development, mitral cells typically extend multiple dendrites to multiple glomeruli and receive heterogeneous OSN inputs. However, during the first postnatal week, they stabilize only one primary dendrite while eliminating others to establish the discrete connectivity. Thus, dendrite pruning is the basis for establishing the discrete receptive field (i.e., the molecular receptive range for odorants) of mitral cells (Figure 2B).

Dendrite pruning is independent of sensory-evoked neuronal activity. Sensory deprivation with naris occlusion does not affect the dendrite pruning process (Fujimoto et al., 2023). Mice deficient for CNGA2 (cyclic nucleotide gated channel alpha 2) are defective in generating odor-evoked depolarization of OSNs and are anosmic, but show normal dendrite pruning of mitral cells (Lin et al., 2000; Fujimoto et al., 2023). Similarly, suppression of spontaneous activity in OSNs did not affect mitral cell pruning (Ma et al., 2014). However, suppression of spontaneous activity in mitral cells disrupted the dendrite pruning process in mitral cells, raising the possibility that spontaneous neuronal activity generated in the olfactory bulb plays a critical role in dendrite pruning (Fujimoto et al., 2023).

## Spontaneous activity in the developing olfactory bulb

Developmental spontaneous neuronal activity was first described in the visual system. Prior to eye opening and visual responses, the developing retina exhibit spontaneous neuronal activity, known as the retinal wave (Meister et al., 1991; Wong et al., 1993; Feller et al., 1996). In particular, the stage II retinal wave, driven by the cholinergic inputs from starburst amacrine cells, is essential for retinotopic map formation, eye-specific segregation, and possibly additional features (Firth et al., 2005; Huberman et al., 2008). *In vivo* imaging in awake neonatal animals confirmed that it is the propagating wave of spontaneous activity (Ackman et al., 2012). Spontaneous activity was also discovered in the auditory system. Supporting cells in the cochlea spontaneously release ATP, which leads to the sporadic activation of nearby hair cells, resulting in striped tonotopic patterns of spontaneous activity, as shown by *in vivo* imaging (Tritsch et al., 2007; Babola et al., 2018). In the whisker region of the somatosensory circuit, spontaneous activity generated in the trigeminal ganglion propagates through the thalamus to the cortex, controlling the whisker-specific patchwork patterns found *in vivo* (Mizuno et al., 2018; Banerjee et al., 2022). While the exact origin of the spontaneous activity is unknown, it is required for the dendritic patterning of L4 neurons in the barrel cortex (Mizuno et al., 2014; Anton-Bolanos et al., 2019).

**Figure 3:**
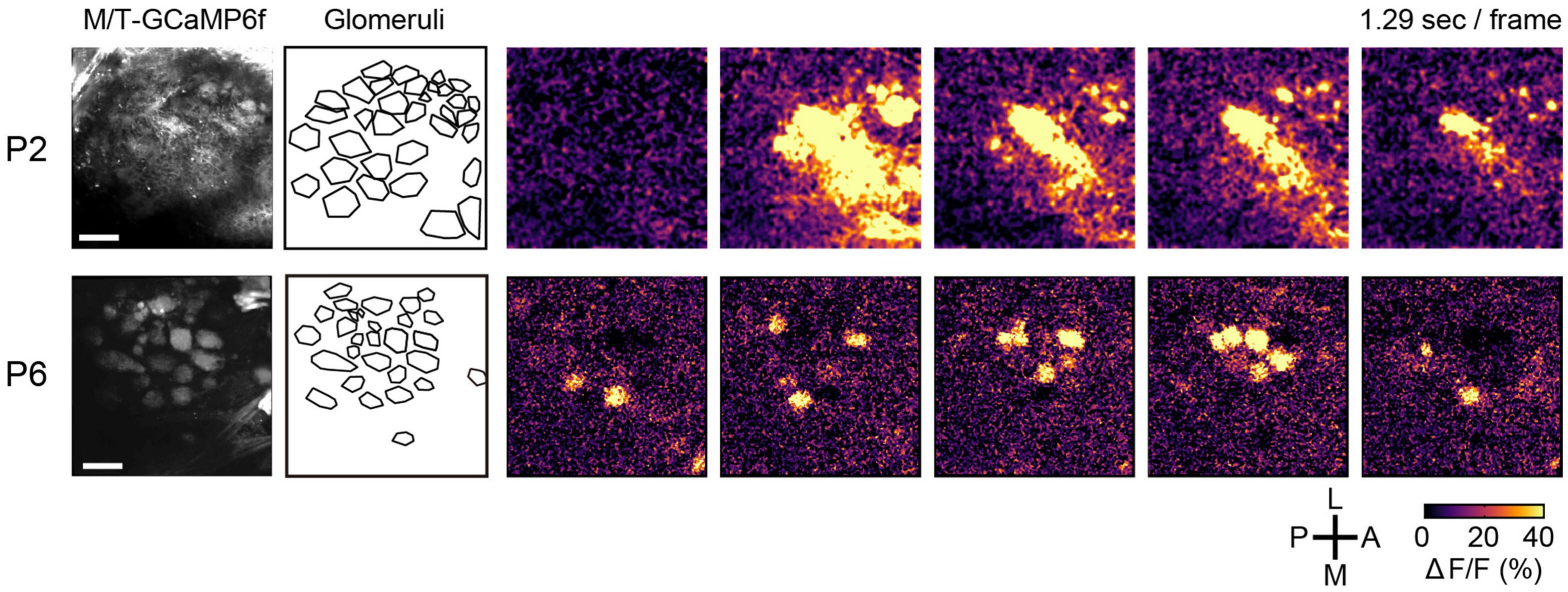
Spontaneous activity in the developing olfactory bulb. Time-lapse images of GCaMP6f (ΔF/F) are shown on the right. During P1-2, the spontaneous activity was propagating waves. At later stages, however, it becomes a glomerulus-specific patchwork pattern. A, anterior; P, posterior; M, medial; L, lateral. Scale bars, 100 μm. Modified from Fujimoto et al. (2023).

To directly observe the spontaneous activity in the olfactory system, Fujimoto et al. performed *in vivo* imaging in awake neonatal animals (Fujimoto et al., 2023). In the early stage (P1-2), the spontaneous activity was a propagating wave that travelled across glomeruli (Figure 3, top). However, at later stages (≥P3), it became glomerulus-specific patchwork patterns (Figure 3, bottom). It was unaffected by sensory deprivation (naris occlusion). The spontaneous activity in the olfactory bulb was independent of the sniff cycles, which is different from the adult animals (Iwata et al., 2017). Furthermore, the spontaneous activity was found in mitral cells, but not in OSNs, suggesting an olfactory bulb origin. The stage-specific spontaneous activity was also observed in the isolated olfactory bulb *ex vivo*, again indicating that it is generated within the olfactory bulb.

Pharmacological experiments indicated that the spontaneous activity is generated by dendro-dendritic glutamatergic transmission (glutamate spillover), a unique feature of the mitral/tufted cells (Fujimoto et al., 2023). Genetic blockade of the dendro-dendritic transmission with tetanus toxin light chain reduced the spontaneous activity and impaired the dendrite pruning process. Dendrite pruning was also blocked by NMDAR knockout in mitral cells, similar to the visual and somatosensory systems. Thus, like the visual, auditory, and somatosensory systems, the remodeling of mitral cell dendrites is driven by spontaneous activity (Fujimoto et al., 2023).

It remains unclear how the patterns of spontaneous activity change from synchronized to patchwork during development. In one scenario, this is simply the result of dendrite pruning. It is also possible that developmental changes in inhibitory synapses decorrelate the spontaneous activity. Indeed, partial blockade of GABA_A_ receptors with Gabazine reversed the patchwork patterns of spontaneous activity at P6 to synchronized patterns *ex vivo* (Fujimoto et al., unpublished data).

## Activity-dependent stabilization of winner dendrites

Due to its characteristic dendritic morphology with a single primary dendrite, the mitral cell is attractive for studying mechanisms of synaptic competition and selective dendrite pruning. Detailed studies of mitral cell remodeling were performed by *in utero* electroporation-based CRISPR-Cas9 screening, tissue clearing-based morphological analysis, and live imaging of acute olfactory bulb slices *ex vivo*.

Aihara et al. investigated possible roles of transmembrane receptors in the dendrite remodeling process (Aihara et al., 2021). They identified BMPR2 (bone morphogenetic protein receptor type 2) as a key player in the activity-dependent dendrite stabilization process. During the remodeling phase, glutamatergic synaptic inputs activate Rac1 through NMDARs. Activated Rac1 then activates PAK [p21 (RAC1) activated kinase], which in turn activates free LIMK (LIM domain kinase) via phosphorylation. Genetic experiments in mitral cells, together with previous biochemical experiments, indicate that ligand-free BMPR2 tethers LIMK at the C-terminal tail, preventing its activation by PAK (Foletta et al., 2003; Aihara et al., 2021). However, the binding of BMPs (bone morphogenetic proteins) to BMPR2 results in the release of LIMK from its C terminus, allowing its phosphorylation by PAK and subsequent phosphorylation of cofilin. While the non-phosphorylated cofilin facilitates the severing of F-actin, the phosphorylated cofilin does not, which leads to the stabilization of the dendrites. Thus, the BMP-BMPR2 signaling “gates” the activity-dependent stabilization of the dendrites: Dendrites receiving both BMP ligands and glutamatergic synaptic inputs are stabilized by F-actin formation (Aihara et al., 2021).

**Figure 4:**
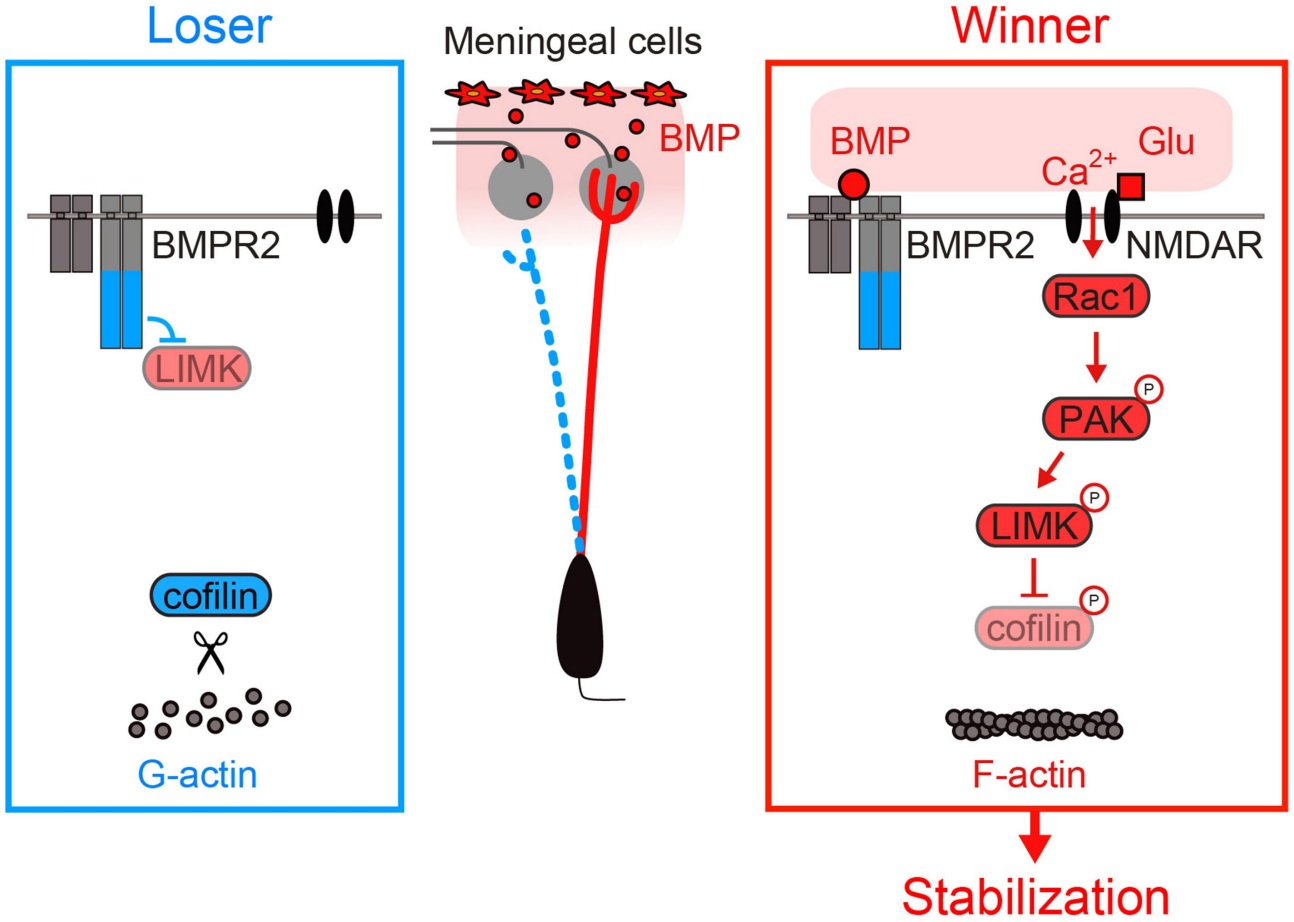
Activity-dependent stabilization of mitral cell dendrites. BMPR2 inhibits LIMK without BMPs. BMPs are secreted from meningeal cells and are most likely enriched in the glomerular layer. LIMK phosphorylation occurs in the presence of both BMPs and NMDAR activation. LIMK phosphorylation leads to dendrite stabilization via increased F-actin formation. Modified from Aihara et al. (2021).

Currently, the protein localization of BMP ligands remains unknown. However, since the *Bmp4* gene is expressed only in the meninges, it is conceivable that BMP is most abundant in the glomerular layer. Indeed, NMDAR activation led to F-actin formation only for dendritic terminals located within the glomerular layer (Aihara et al., 2021). Once a dendrite is stabilized and forms a tuft structure within the glomerulus, it should receive more BMP signals and more NMDAR-dependent Rac1 signals. In this way, the dendrites with rich synaptic inputs become richer. The positive feedback mechanisms involving BMPR2 and NMDARs may be critical for establishing a strong primary dendrite during the remodeling process (Figure 4).

## Activity-dependent pruning of loser dendrites: the punishment signals and local protection signals

It is well known that NMDAR knockout impairs both the stabilization of winner synapses and the pruning of loser synapses. NMDAR-deficient mitral cells fail to prune dendrites (Fujimoto et al., 2023). How can NMDARs coordinate both stabilization and pruning of dendrites within a mitral cell?

Fujimoto et al. investigated possible roles for Rho family small GTPases and found that RhoA is essential for NMDAR-dependent pruning of mitral cell dendrites (Fujimoto et al., 2023). Triple knockout of RhoA/B/C impaired dendrite pruning. A pruning defect seen with NMDAR knockout was rescued by RhoA overexpression. Overexpression of constitutively active RhoA results in overpruning of dendrites. Indeed, RhoA is known to facilitate neurite retraction (Luo, 2000). However, this is puzzling because RhoA must be activated in loser dendrites, whereas NMDAR activation should be highest in winner dendrites with rich glutamatergic synaptic inputs. How can NMDARs activate RhoA only in the distant losing dendrites?

**Figure 5:**
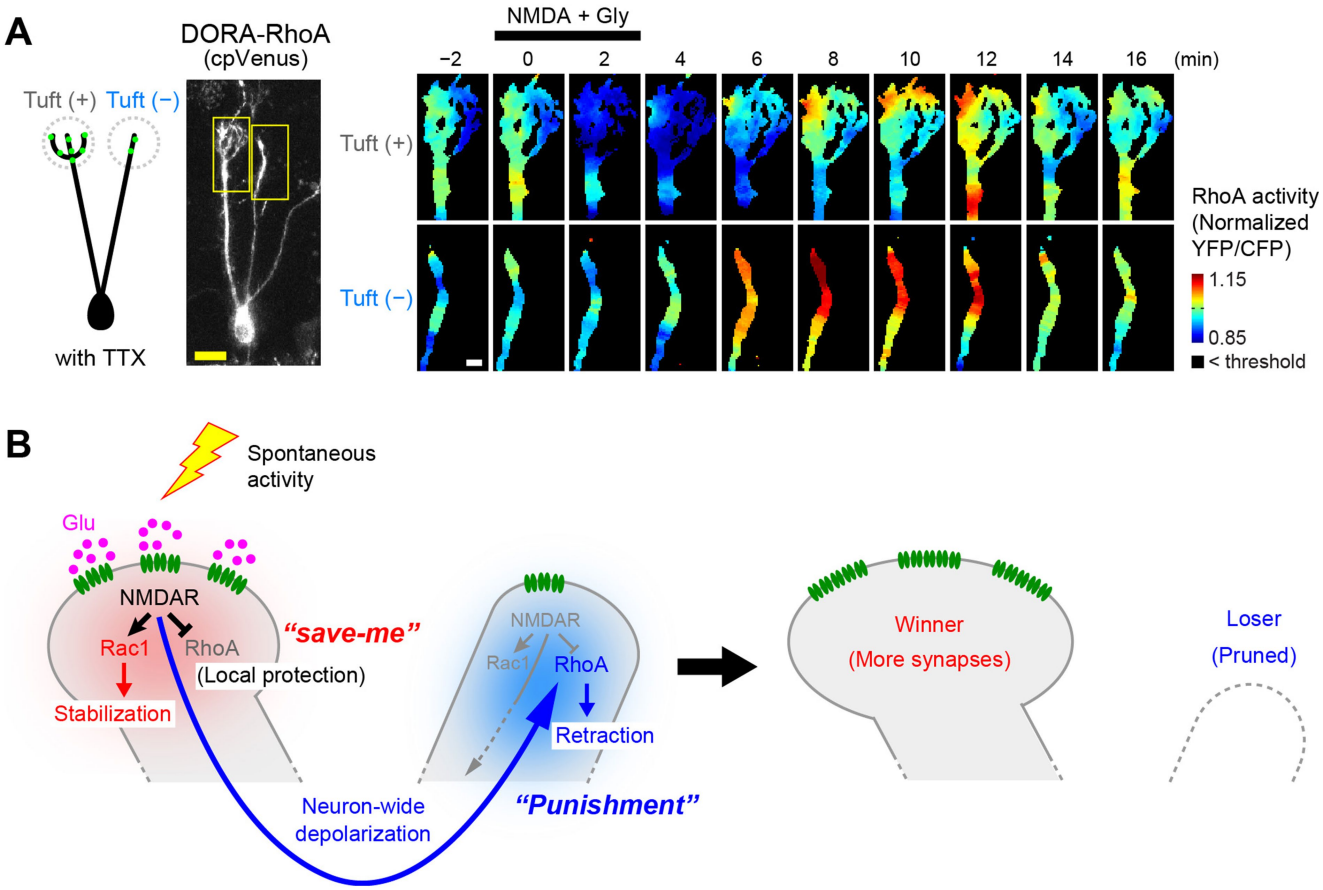
Activity-dependent pruning of loser dendrites by RhoA regulation. **(A)** FRET imaging of RhoA activity upon NMDA stimulation in olfactory bulb slices (P3). Glutamatergic synapses (green dots in the cartoon) are rich in tuft(+) dendrites but poor in tuft(−) dendrites. Time-lapse images of RhoA activity (normalized YFP/CFP ratio) are shown on the right. Upon NMDA stimulation, NMDARs are activated mainly in tuft(+) dendrites. In this case, RhoA activity is reduced in the tuft(+) dendrites but activated in tuft(−) dendrites, most likely via neuronal depolarization. Scale bars, 20 μm (left) and 5 μm (right). **(B)** A proposed model for the NMDAR-dependent RhoA regulation and dendrite pruning. NMDARs locally suppress RhoA to protect the activated dendrite from pruning. On the other hand, subsequent neuronal depolarization globally activates RhoA to facilitate pruning of non-protected dendrites. Local suppression of RhoA serves as a *“save-me”* signal, whereas the remote activation of RhoA serves as a *“punishment”* (lateral inhibition) signal. Modified from Fujimoto et al. (2023).

Using FRET imaging of the RhoA sensor in acute olfactory bulb slices, Fujimoto et al. discovered that NMDARs play a dual role in regulating RhoA (Fujimoto et al., 2023). When NMDARs are activated in a dendrite, RhoA is inhibited in the vicinity of the activated NMDARs (Figure 5A, top). At the same time, RhoA is activated in the other dendrites and somata (Figure 5A, bottom). Thus, the NMDAR-dependent local signals and remote signals have opposite effects on RhoA activity. When mitral cells were depolarized by high K^+^ stimulation, RhoA was activated in all dendrites, suggesting that the remote signals are mediated by neuronal depolarization.

NMDAR-dependent local suppression of RhoA antagonizes the pruning signals (i.e., RhoA activation) and protects the activated dendrite from pruning, thus acting as the local protection signals (*“save-me”* signals). On the other hand, depolarization-dependent global activation of RhoA, leading to retraction of dendrites, acts as a lateral inhibition signal for non-protected dendrites. This may be the direct evidence for the long-hypothesized *“punishment”* signals for synapse elimination (Lichtman and Colman, 2000; Figure 5B).

Currently, the nature of the remote and local signals via NMDARs remains elusive. Most likely, the NMDARs may locally activate RhoGAPs to suppress RhoA, whereas NMDAR-dependent depolarization may globally activate RhoGEFs to facilitate dendrite retraction. It will be important to determine which specific RhoGAPs and RhoGEFs are involved and how NMDARs differentially control the different pathways for local protection and global pruning, respectively.

## How can the out-of-sync inputs facilitate synapse elimination?

**Figure 6:**
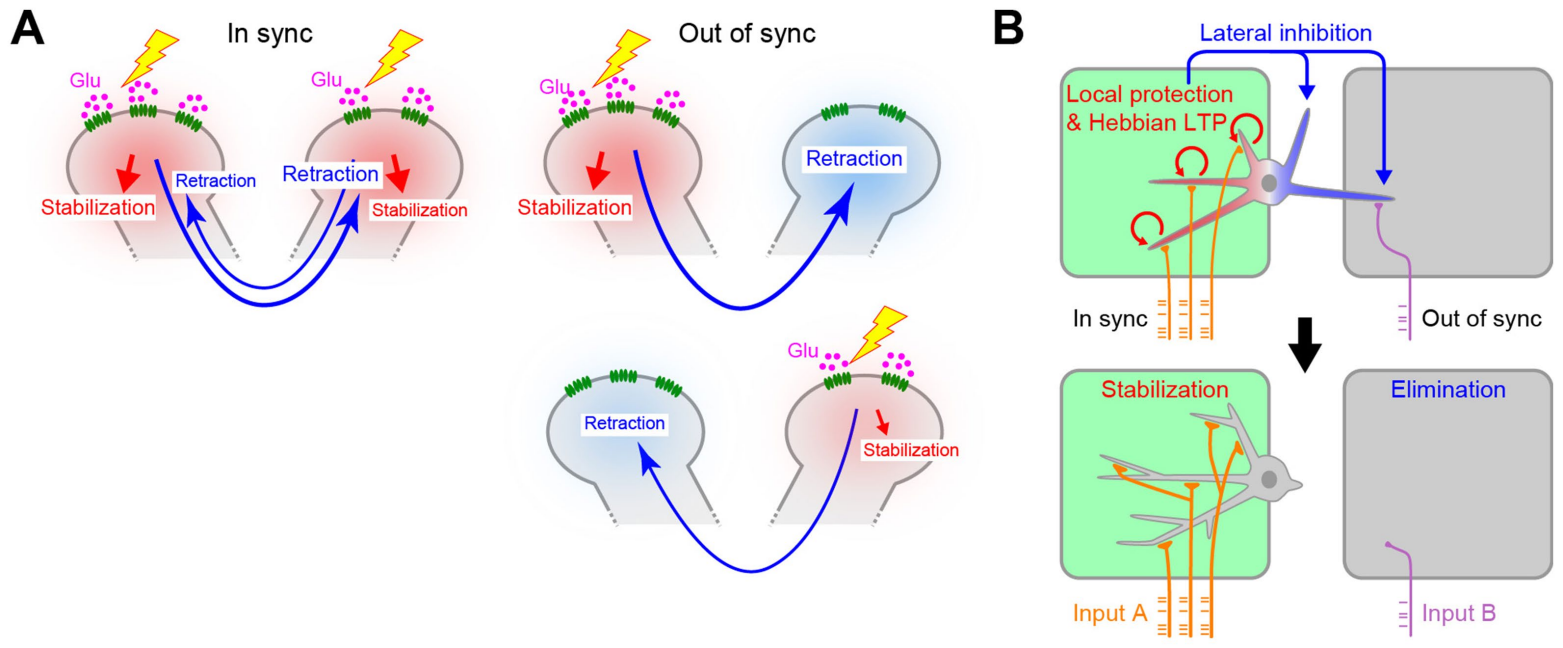
Role of timing in activity-dependent dendrite pruning. **(A)** Asynchronous synaptic inputs are more efficient for lateral inhibition between dendrites. Synchronous synaptic inputs would protect all the synapses by RhoA suppression. As a result, lateral inhibition signals cannot efficiently facilitate dendrite pruning. The out-of-sync inputs allow dendrites to send lateral inhibition signals to non-protected dendrites for dendrite pruning, even when two dendrites initially have a similar number of synapses. Depending on the pattern of synaptic inputs, even the weaker one can become the winner. **(B)** In the L4 neurons in the barrel cortex, multiple dendrites within the same hollow receive synchronous synaptic inputs and are protected together. Only dendrites outside of the major hollow are pruned. Schemas from Fujimoto et al. (2023).

This model also explains why the asynchronous synaptic inputs found later in development facilitate synapse elimination. Before the synaptic competition, different dendrites are likely to have similar numbers of postsynaptic sites. If synaptic inputs to all dendrites were synchronous, all dendrites would be protected together. However, if the synaptic inputs were asynchronous across dendrites, they would send the lateral inhibition signals to the non-protected dendrites. By repeating the lateral inhibition signals, driven by the spontaneous neuronal activity of the glomerulus-specific patchwork patterns, the subtle difference in synaptic strength would increase over time, eventually eliminating all but just one primary dendrite (Figure 6A).

Fujimoto et al. further demonstrated that RhoA signaling is also essential for whisker-specific dendritic patterning in L4 neurons in the barrel cortex (Fujimoto et al., 2023). The difference here is that multiple dendrites remain for the barrel cortex, whereas only one primary dendrite remains for the mitral cells. Again, the spatiotemporal patterns of the activity explain the difference between these two systems. In the olfactory bulb, dendrites in different glomeruli receive asynchronous synaptic inputs, especially at a late stage (Fujimoto et al., 2023). This leads to lateral inhibition, leaving only one primary dendrite per mitral cell. However, in the barrel cortex, multiple dendrites connected to the same hollow receives synchronous synaptic inputs driven by the whisker-specific patchwork spontaneous activity (Mizuno et al., 2018). Therefore, these dendrites are all protected by synchronous RhoA suppression. Only those that are non-protected at the time of the synchronous synaptic inputs (i.e., outside the hollow) are pruned by the RhoA activation (Figure 6B).

## Activity-dependent synapse elimination in other model systems and during learning process

So far, the role of NMDAR—RhoA signaling has only been tested for mitral cells and L4 neurons in the barrel cortex. There are some studies suggesting that NMDARs are also involved in synapse elimination in neuromuscular junction and climbing fiber—Purkinje cell synapses (Rabacchi et al., 1992; Personius et al., 2016); however, they may need to be carefully evaluated because NMDARs are not functional at mature stages in these synapses. It will be interesting to test whether RhoA is involved in synapse elimination in these cells, as the postsynapses are not located at dendrites during the synaptic competition. Nevertheless, similar mechanisms should be at work in these cells, and synaptic competition in the postsynaptic cell is central to the choice between stabilization and elimination of presynaptic axons.

Recent studies have shown how the axon pruning is regulated downstream of the hypothetical punishment signals. Using callosal neurons as a model system, Yasuda et al. reported that the JAK2/STAT1 pathway is activated in inactive axons, leading to axon pruning (Yasuda et al., 2021). It is currently unknown how JAK2 is activated. In one scenario, it may be activated upon loss of postsynaptic structure. Alternatively, it may be activated by the retrograde signals downstream of the hypothetical punishment signals (e.g., RhoA) (Nagappan-Chettiar et al., 2023). Semaphorins are candidate molecules for the retrograde signaling at the climbing fiber – Purkinje cell synapses (Uesaka et al., 2014). At the neuromuscular junction, a microtubule severing protein, spastin, is involved in the branch-specific axon pruning in motor neurons, possibly downstream of JAK2/STAT1 (Brill et al., 2016).

Neurite pruning is also studied in the injury models and during fly metamorphosis (Luo and O'Leary, 2005). In the injured axons, caspase-dependent calpastatin depletion leads to axon degeneration (Yang et al., 2013). During metamorphosis in *Drosophila*, thinning of proximal dendrites and subsequent compartmentalized Ca^2+^ transients lead to degeneration of the severed dendrites in sensory neurons, mediated by caspases and calpains (Kanamori et al., 2013; Kanamori et al., 2015). However, these mechanisms are not dependent on synaptic competition. The underlying molecular mechanisms should be fundamentally different from those discussed above.

Synapse elimination is widespread not only during development, but also during activity-dependent plasticity in the adult. *In vivo* calcium and morphological imaging suggest the rules for the synaptic potentiation versus elimination. In the motor cortex, asynchronous activity with nearby synapses or the somatic activity leads to synapse depotentiation and elimination, suggesting similar forms of synaptic competition during the learning process (Cichon and Gan, 2015; Hedrick et al., 2024).

## Role of glia in synapse elimination

Microglia are gaining attention as a possible key player in synapse elimination during development and under pathological conditions. For example, during the activity-dependent remodeling of retinal ganglion cell axons, microglia are reported to engulf their synapses and mediate synaptic pruning (Stevens et al., 2007; Schafer et al., 2012; Faust et al., 2021). It has also been proposed that the *“eat-me”* signals presented by the loser synapses are engulfed by microglia (Nagappan-Chettiar et al., 2023). However, Niiyama et al. found that microglia are dispensable for the activity-dependent dendrite pruning in mitral cells and L4 neurons in the barrel cortex (Niiyama et al., 2023): Pharmacological depletion of microglia had no effect on the dendrite pruning process. A more recent study also failed to find a role for microglia in activity-dependent development of the visual circuit (Brown et al., 2024). Studies in other circuits have also failed to find evidence for a role for microglia in developmental synapse elimination (O’Keeffe et al., 2025; Surala et al., 2024). Studies of climbing fiber—Purkinje cell synapses also failed to find evidence for direct synaptic engulfment by microglia (Nakayama et al., 2018). It remains possible that other types of glia, astrocytes and oligodendrocyte precursors (OPCs), have functionally redundant roles in synapse elimination, as they have also been reported to engulf synapses (Chung et al., 2013; Auguste et al., 2022; Buchanan et al., 2022). Nevertheless, it appears that the role of microglia in synapse elimination have been overstated in the earlier studies (Faust et al., 2021).

As discussed above, the activity-dependent synapse elimination is the result of synaptic competition in postsynaptic cells. In the developmental synaptic competition, the decision of winner versus loser synapses should be made in postsynaptic cells. Therefore, the contribution of glia, if any, should come after the decision to eliminate the synapses by the neurons themselves.

## Concluding remarks

In this review, I summarized our current understanding of synaptic competition with a particular focus on dendrite pruning of mitral cells in the olfactory bulb. Mitral cells have become a useful model system to study developmental synaptic competition due to their unique morphological features and the availability of various genetic and imaging tools (*ex vivo* and *in vivo*). As for the activity-dependent stabilization of winner dendrites, NMDAR-dependent F-actin formation and subsequent positive feedback loops of stabilization signals (BMP and glutamatergic inputs) are critical (Figure 4). In contrast, pruning of loser dendrites is mediated by a combination of NMDAR-dependent local protection signals (*“save-me”* signals, i.e., RhoA suppression) and neuron-wide activation of lateral inhibition signals (*“punishment”* signals, i.e., RhoA activation) (Figure 5B).

The remaining important question is then how synaptic inputs via NMDARs control distinct signaling pathways for stabilization, local protection, and lateral inhibition. These are most likely driven by local Rac1-GEF activation, local RhoA-GAP activation, and global RhoA-GEF activation, respectively. Investigating the molecular and cellular logic behind the spatiotemporal regulation of these distinct signaling pathways should clarify the long-standing mysteries of synapse elimination, an important aspect of neural development.

The synaptic competition discussed here is a form of heterosynaptic plasticity in which inputs to one synapse influence the plasticity of other synapses (Chater and Goda, 2021; Jenks et al., 2021). With the development of fluorescent labeling and large-scale imaging techniques, it is becoming easier to capture the full picture of the synaptic organization at the whole-neuron scale. These techniques, together with genetic studies of molecular mechanisms, facilitate our understanding of how a neuron modulates thousands of synaptic inputs to acquire mature neuronal functions during development, a long-standing question since the seminal work by Hubel and Wiesel (1962).
